# Herpes zoster incognito: an immunohistochemical diagnosis^☆^^☆☆^

**DOI:** 10.1016/j.abd.2019.07.011

**Published:** 2020-03-19

**Authors:** Gianluca Nazzaro, Stefano Veraldi

**Affiliations:** Department of Pathophysiology and Transplantation, Università degli Studi di Milano, Fondazione IRCCS Ca’ Granda Ospedale Maggiore Policlinico, Milan, Italy

Dear Editor,

A 60-year-old woman presented with a 1 week history of an erythematosus and edematous plaque on her scalp. The lesion, 2.5 cm in diameter, was associated to mild pain (Fig. 1). Histopathological examination showed a massive inflammatory infiltrate in the dermis, especially surrounding sebaceous glands and responsible of oedema in the papillary dermis with initial dermo-epidermal vescicle formation (Fig. 2A). In the suspect of a herpetic infection, immunohistochemistry was performed, revealing negativity for Herpes Simplex virus (HSV) and positivity for Varicella Zoster virus (VZV) (Fig. 2B). Our diagnosis was therefore herpes zoster incognito.

**Figure 1:**
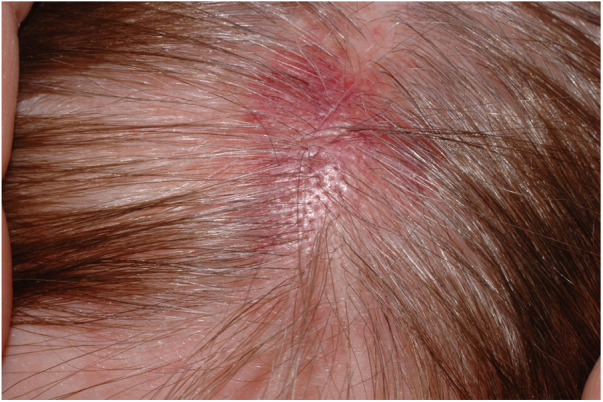
Erythematous plaque with recent onset at the top of a 60 year-old woman.

**Figure 2:**
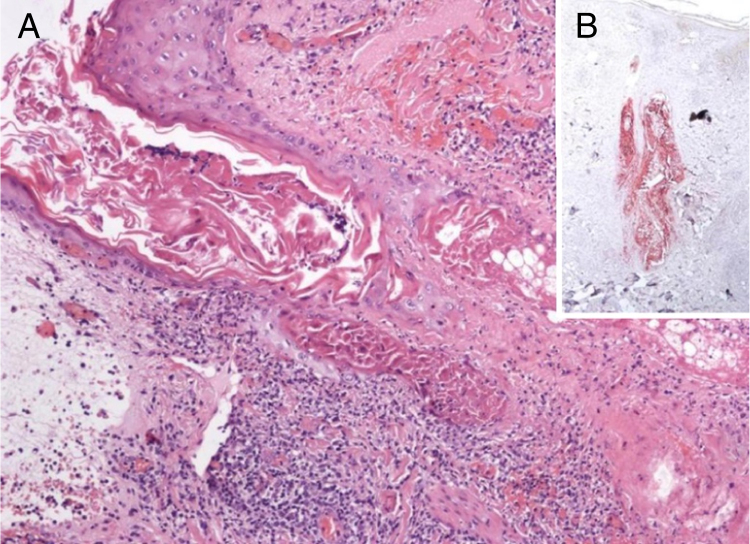
A, Inflammatory infiltrate surrounding follicular epithelium and sebaceous gland. Initial bullous detachment at the dermo-epidermal junction (Hematoxylin & eosin, ×40). B, The primary involvement of the skin is on the sebaceous epithelium, as shown by immunohistochemistry specific for varicella zoster virus (Hematoxylin & eosin, ×40).

Herpes zoster (HZ), due to the reactivation of VZV, present in a latent state in sensory ganglia, can remain inactive for decades or also for the whole life. Essential condition is a previous contact, usually during infancy, with VZV. HZ is characterized by unilateral erythematosus-vesicular rash and a localized pain. Nevertheless, especially at the onset of an eruption or in forms considered as abortive, when lesions are macules, papules, and plaques, clinical diagnosis may be challenging. Microscopical examination can be used to confirm infection by herpesviruses, but sometimes typical signs such as multinucleated epithelial cells or ghosts of them are not encountered in a specimen. The term “herpes incognito” (from Latin, meaning not recognizable) has therefore been introduced.^1^

The virus is transported from dorsal root or trigeminal ganglia via myelinated nerves which terminate at the isthmus of hair follicles. Consequently, the primary involvement of the skin is on follicular and sebaceous epithelium,^2^ as shown in the case presented. Spread of infection to the epidermis follows. This clue, that is pathognomonic of HZ and is not found in herpes simplex, can be easily confirmed by immunohistochemistry. This histological method, that can be used to distinguish the viral aetiology in tricky causes,^3^ demonstrates that HSV affects primarily the epidermis and the upper portions of follicles only occasionally but never sebaceous epithelium or nerves. In fact, VZV spreads preferentially from dermal nerves to folliculosebaceous units and thence to the epidermis. Nevertheless, the reason why recurrent HSV infection primarily targets the epidermis, in contrast to HZV, which is preferentially directed to folliculosebaceous units, is not fully understood.

In a study involving 75 patients with a clinical differential diagnosis of herpetic infections,^4^ HZ was misdiagnosed as HSV infection in 30% of the cases as the clinicians were in difficult when vesicles were absent. From a histological point of view, herpetic folliculitis was detected in 28% of HZ, while it was not encountered in herpes simplex infections.

In conclusion, HZ may present with clinical variants, such as purpuric or hemorrhagic^5^ in patients in antiplatelet or anticoagulant therapy and, gangrenous, bullous or disseminated HZ in immunocompromised patients. We described a case of herpes incognito, an underreported clinical variant of HZ, representing its abortive form, with distinctive clinical and histological features.

## Financial support

None declared.

## Authors’ contributions

Gianluca Nazzaro: Approval of the final version of the manuscript; elaboration and writing of the manuscript; obtaining, analysis, and interpretation of the data; effective participation in research orientation; critical review of the literature; critical review of the manuscript.

Stefano Veraldi: Approval of the final version of the manuscript; critical review of the literature.

## Conflicts of interest

None declared.
